# An MIMO Radar System Based on the Sparse-Array and Its Frequency Migration Calibration Method

**DOI:** 10.3390/s19163580

**Published:** 2019-08-17

**Authors:** Yue Ma, Chen Miao, Yangying Zhao, Wen Wu

**Affiliations:** Ministerial Key Laboratory of JGMT, Nanjing University of Science and Technology, Xiao Ling Wei200#, Nanjing 210094, China

**Keywords:** TDM, MIMO, DOA, Sparse-array, frequency migration, time stretching transform

## Abstract

In this paper, a Multiple Input Multiple Output (MIMO) radar system based on a sparse-array is proposed. In order to reduce the side-lobe level, a genetic algorithm (GA) is used to optimize the array arrangement. To reduce the complexity of the system, time-division multiplexing (TDM) technology is adopted. Since the signals are received in different periods, a frequency migration will emerge if the target is in motion, which will lead to the lower direction-of-arrival (DOA) performance of the system. To solve this problem, a stretching transformation method in the fast-frequency slow-time domain is proposed, in order to eliminate frequency migration. Only minor adjustments need to be implemented for the signal processing, and the root-mean-square error (RMSE) of the DOA estimation will be reduced by about 90%, compared with the one of an uncalibrated system. For example, a uniform linear array (ULA) MIMO system with 2 transmitters and 20 receivers can be replaced by the proposed system with 2 transmitters and 12 receivers, achieving the same DOA performance. The calibration formulations are given, and the simulation results of the automotive radar system are also provided, which validate the theory.

## 1. Introduction

Multiple-input multiple-output (MIMO) technology [1,2,3] has been widely used in imaging, detection and estimation domain. In recent years, it has also been used extensively in automotive radars [4]. Imaging is an important application of MIMO radar; Ciuonzo [5] designed and analyzed the computational wideband time-reversal (TR) imaging algorithms, while Devaney [6] employed the methods of time-reversal imaging to image and locate the point targets obscured by an inhomogeneous background medium such as the ionosphere or foliage. Direction-of-arrival (DOA) estimation is the key technology of a MIMO system. In [7], Cao et al. applied the tensor-based methods to DOA estimation in a MIMO radar. Ciuonzo et al. studied the performance of multiple signal classification (MUSIC) in computational time-reversal applications [8]. Zhou et al. proposed a novel virtual array interpolation-based algorithm for coprime array DOA estimation [9]. Nowadays, the sparse-array-based [10] MIMO radar is becoming the trend of the radar system. With the same array aperture, but fewer antenna elements, the sparse-array can achieve the same resolution, but reduces the complexity, size and weight of the system. Due to the fact that the virtual array contains more virtual sensors than physical ones, it effectively enhances the performance of degrees of freedom (DOFs) [11]. Compared with the uniform linear array (ULA), both beamforming and DOA based on sparse-array [12] have more advantages, but considering the large influence on the antenna side-lobe caused by the sparse-array, many optimization methods of the sparse-array emerge.

The particle swarm optimization (PSO) [13] starts from the random arrangement of the sparse-array, selects a variable (such as the side-lobe of radiation) as the fitness function, and then searches for the optimal solution through the iterative search process. However, PSO is prone to fall into the local optimal, leading to the uncertainty of the optimal solution. The minimum redundancy [14] is an optimization of array configuration by the exhaustion method, but if a little more elements optimized, it will be a massive calculation, and it is not useful for processing coherent signals. Compared with them, the genetic algorithm (GA) [15] can obtain the optimal global solution and optimize a large number of elements, but require less computation. In this paper, GA is adopted for its simplicity and high efficiency.

To separate different transmitter signals at the receiver, Time-division transmission (TDM), frequency division transmission, code division multiplexing and other methods are usually used. Among them, TDM [16] is the simplest choice to realize the MIMO radar system on hardware, and it is also widely used in practical engineering. Compared with the pulse radar system, the linear- frequency-modulated continuous-wave (LFMCW) [17] system’s anti-jamming performance is better, and it is transmitted by TDM in this system. Because of the time division mode of TDM system, the Doppler Effect causes additional phase shifts for a motion target [18]. Guetlein [19] and Rambach [20] proposed modulation schemes to eliminate the phase shift by compensating the target, but their strategies only considered the scenario for a single target. In [21], sparse reconstruction is applied to deal with the phase shifts, but it takes a lot of steps and calculations. A phase error correction method based on DFT is proposed in [22]. While all of the above methods [18,19,20,21,22] are focused on eliminating the phase shift. In this paper, a migration caused by frequency is found existing in the system for the high-speed target detection (such as in automotive radar, the high speed refers to 72 km/h to 300 km/h). There is a term in echo signal formulation which depends upon fast time, velocity and slow time. This term will have an impact on FFT results, especially for a multiple transmitter system, which leads to the deviation of the measured target frequency point, i.e., frequency migration. Due to its existence, the DOA performance of the system to detect motion targets will be degraded. Therefore, a stretching transformation implementing in a fast-frequency slow-time domain is proposed in this paper, which does not require any additional hardware effort, and has only few processing steps. Monte Carlo experiments are carried out in different SNR, and simulation results show that the DOA estimation performance of the system after calibration improved greatly. A low cost and high precision MIMO radar system based on a sparse-array is presented in this paper, and the proposed calibration method will help improve the DOA performance of the radar systems using the TDM.

This paper is organized as follows: Section 2 explains the principle of the TDM MIMO radar system based on the sparse-array, then the arrangement of sparse-array is optimized by GA to obtain high performance. In Section 3, the signal model and working principle of the proposed system is introduced. The DOA parameters are simulated, which exhibits that the precision and angular resolution of the system will decrease when detecting a motion target without frequency migration calibration. Then the method of signal calibration processing is introduced, and the simulation is performed to verify the effectiveness of the calibration. After that, the accurate DOA results of the proposed system under different SNR values are obtained by the simulation with the calibration steps. Finally, conclusions are drawn in Section 4.

Notations: In this paper, for a matrix A, $A^{T}$ denotes its transpose, $\left|A^{T}\right|$ denotes its absolute value, ⊗ represents a Kronecker Product. The bold upper case letters are matrices, and the italic letters show scalar variables. For a function f(x), min{f(x)} represents the minimum value of f(x).

## 2. Sparse-Array Optimization for MIMO Radar

### 2.1. MIMO Radar Based on Sparse-Array

**Figure 1:**
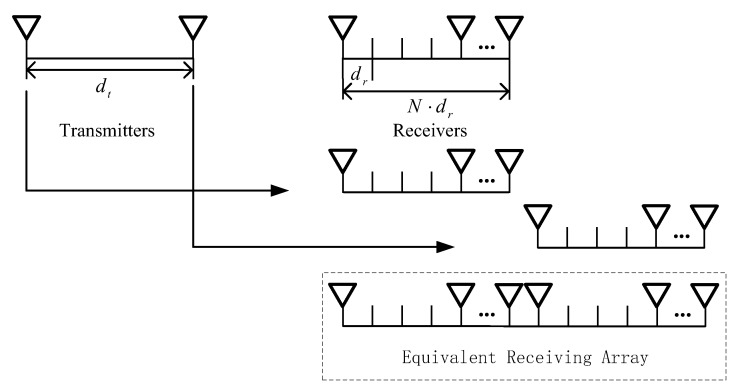
The structure of sparse-array Multiple Input Multiple Output (MIMO) radar.

The structure of a sparse-array MIMO radar is shown in Figure 1. Assume that the system consists of M transmitters (Tx) with the array spacing $d_{t}$, and its receiver array (Rx) has a total of G receivers, which are sparsely distributed on *N* grid points. If the spacing between two grid points is $d_{r}$, then $d_{t}=N\cdot d_{r}$. Set the first receiver as the reference element and the distance between the *n*th receiver and the first one is $d_{rn},n=0,1,\ldots ,G-1$.

As shown in Figure 1, the Tx and Rx can be equivalent to a virtual array containing M×G elements with an array aperture of M×N. The MIMO system takes fewer array elements than a conventional system, such as the phased array system, but with larger array aperture. The ideal signal model of the radar system can be expressed as:

(1) X(t)=As(t)+n(t)

where s(t) is the echo signal, n(t) is the Gaussian white noise. Assuming the steering vector of the supposed transmitting antennas and receiving antennas as $a_{t}(t)$ and $a_{r}(t)$ along direction θ, A is the steering vector of the equivalent receiving array. They can be represented by Formula (2)–(4) as follows:

(2) $$a_{t}(\theta )={\left[1,e^{\frac{-j2\pi f_{0}\cdot Nd\sin\theta }{c}},e^{\frac{-j2\pi f_{0}\cdot 2Nd\sin\theta }{c}},\ldots ,e^{\frac{-j2\pi f_{0}\cdot (M-1)\cdot Nd\sin\theta }{c}}\right]}^{T}$$

(3) $$a_{r}(\theta )={\left[1,e^{\frac{-j2\pi f_{0}d_{r1}\sin\theta }{c}},e^{\frac{-j2\pi f_{0}2d_{r2}\sin\theta }{c}},\ldots ,e^{\frac{-j2\pi f_{0}(N-1)d_{r(G-1)}\sin\theta }{c}}\right]}^{T}$$

(4) $$A=a_{t}(\theta )⊗a_{r}(\theta )={\left[1,e^{\frac{-j2\pi f_{0}d_{r1}\sin\theta }{c}},\ldots ,e^{\frac{-j2\pi f_{0}(mnd+d_{r(G-1)})\sin\theta }{c}},\ldots ,e^{\frac{-j2\pi f_{0}\cdot [(M-1)\cdot Nd+(N-1)d_{r(G-1)}]\sin\theta }{c}}\right]}^{T}$$

where m=0,1,…,M−1; n=0,1,…,G−1; $f_{0}$ is the carrier frequency, c is the velocity of light.

### 2.2. Optimization Design of the Sparse-Array MIMO Radar

Assuming that the virtual equivalent array is illuminated by the plane wave with an incident angle of θ, the radiation pattern of the system can be obtained:

(5) $$F\left(\theta \right)=\left|A^{T}\right|=\left|\left[1,e^{\frac{-j2\pi f_{0}d_{r1}\sin\theta }{c}},\ldots ,e^{\frac{-j2\pi f_{0}(mnd+d_{r(G-1)})\sin\theta }{c}},\ldots ,e^{\frac{-j2\pi f_{0}\cdot [(M-1)\cdot Nd+(N-1)d_{r(G-1)}]\sin\theta }{c}}\right]\right|$$

Compared with the conventional ULA, the side-lobe level of the radar system based on sparse-array becomes higher. To solve this problem, a GA is used to optimize the arrangement of sparse-arrays [15]. Suppose the system has two transmitters, and $d_{r}$ is constant. According to Formula (5), the receiving array spacing is the most important factor affecting the performance of the system’s radiation pattern. The purpose of GA is to obtain the system radiation pattern with the lowest side-lobe by optimizing the array arrangement. The steps of GA can be described in Algorithm 1.

**Algorithm 1** The optimize of sparse-arrays’ arrangement by GA**Step 1 Initialization:** Initializing the individual chromosome, which represents the sparse-array arrangement. The length of the individual chromosome is initialed as I (that is, the number of grid points N). The existence or nonexistence of each gene in a chromosome is defined by binary code 1 and 0. Initializing the number of array elements G to randomly generate the initial population $N_{p}$, initial crossover probability as $P_{c}$, initial mutation probability as $P_{m}$, and initial iteration times as $N_{t}$. s is the probability of random generation of each gene.**Step 2 Fitness assignment**: Substituting each individual chromosome into the fitness function to calculate the corresponding fitness value. The peak side lobe level in the radiation pattern is taken as the fitness function, which is given as $f\left(i\right)=\min\left\{SL_{p-i}\right\}$, where $SL_{p-i}$ is the peak side-lobe level of the *i*th optimized array.**Step 3 Selection:** The optimal fitness value and chromosome of each generation were both reserved, and the rest were selected according to the Roulette Wheel Selection Method to pass on to the next generation.**Step 4 Crossover**: Crossover was carried out in this step, and it will occur if $s>P_{c}$; otherwise, no crossover will occur.**Step 5 Mutation:** Mutation was carried out in this step, and it will occur if $s>P_{m}$; otherwise, no mutation will occur. **Step 6 Termination conditions determine:** The iteration times and the change rate of the optimal peak side-lobe level were taken as the termination conditions. If one of the termination conditions was satisfied, then the individuals with the best fitness of a certain generation can be obtained as the global optimal solution, which is the optimal arrangement of sparse-array elements; otherwise, go back to Step 2 and repeat.

In the process of crossover and mutation, the array aperture and sparse rate (that is, the number of array elements) should be kept unchanged, so the position of the first and last elements on raster points should be fixed. In addition, the selected position should be represented as the number of “1” in the gene if the crossover does not happen. If the mutation position changes from “0” to “1”, another “1” position should be selected and reversed, and vice versa.

Next, the computational complexity of Algorithm 1 is discussed. The major computations are in Step 2, and this is due to the calculation of the fitness function, whose computation complexity is about $O\left(\pi N_{p}\right)$. The number of iterations of Algorithm 1 depends on the termination condition, which means it will not exceed $N_{t}$, so the overall complexity of Algorithm 1 is no more than $O\left(\pi N_{p}N_{t}\right)$.

**Figure 2:**
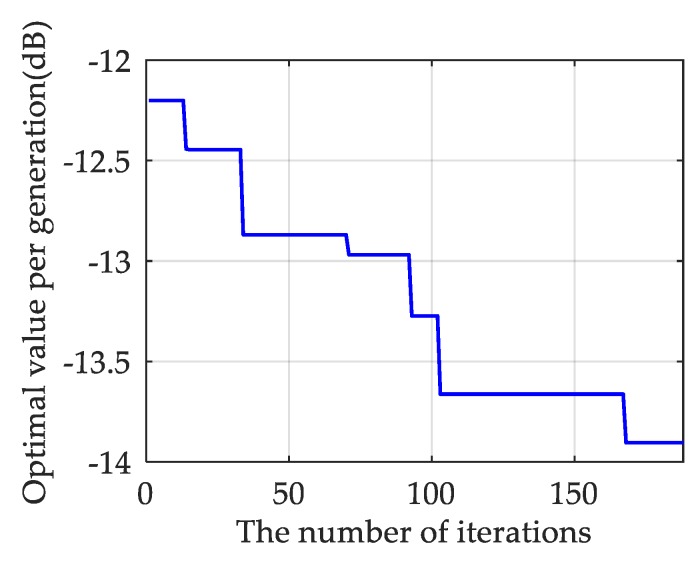
The optimization process of genetic algorithm.

Based on the above steps, set I=20, G=12, $N_{p}=40$, $P_{c}=0.8$, $P_{m}=0.05$, $N_{t}=400$. The iterative process of GA is shown in Figure 2.

**Figure 3:**
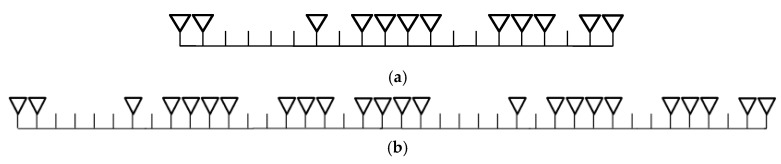
(**a**) The optimized receiver array arrangement; (**b**) Virtual equivalent array of MIMO system.

After 188 iterations, the peak side-lobe level of −13.9dB is obtained and the optimized result is shown in Figure 3a. The structure of the equivalent virtual array arrangement of the sparse-array MIMO system is shown in Figure 3b. The spacing between grid points is $d_{r}=\lambda /2$, where λ is the wavelength of the signal.

### 2.3. Analysis of Optimization Results

**Figure 4:**
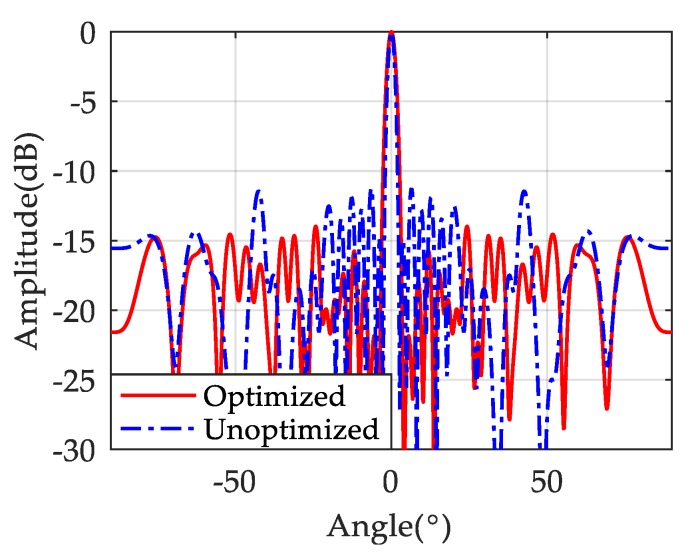
The radiation pattern of optimized and un-optimized array.

The optimized radiation pattern of the system compared with the ones of un-optimized array are shown in Figure 4, it can be seen that the maximum value of the side lobe is significantly decreased, with the main lobe almost unchanged.

Considering an automotive radar, this paper is mainly focused on MIMO and other technologies to improve the DOA performance. The receive array of the system is optimized as the arrangement shown in Figure 3a. In order to verify the performance of the sparse-array MIMO radar system, the simulation is carried out. 

**Figure 5:**
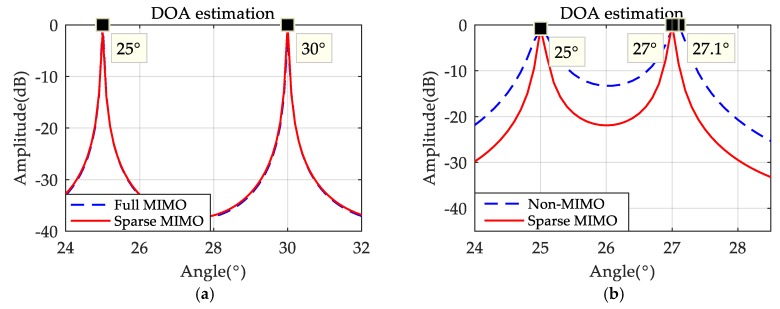
(**a**) direction-of-arrival (DOA) results under the same array aperture; (**b**) DOA results under the same array elements.

System detection angles range from 0° to 180°, and suppose there are two static targets and their angles are $\theta_{1}\;=\;25{}^{\circ}$, $\theta_{2}\;=\;30{}^{\circ}$ respectively. In addition, the detection range of automobile radar is generally between 0 and 400 m, and the distance of the two targets is assumed to be 350 m in the following simulation. The result of DOA by MUSIC [16,23] is shown in Figure 5a, which indicates that, with the same array aperture, DOA performance of full array MIMO radar and of sparse-array MIMO radar are similar. Then, the sparse-array MIMO radar is compared with a radar with 24 uniformly-distributed array elements, and the angle of the second target was changed to $\theta_{2}\;=\;27{}^{\circ}$, where the result of DOA is shown in Figure 5b. It is obvious that the DOA performance of a sparse-array MIMO system is better. Although the number of ULA’s elements is the same as the number of virtual elements in a sparse MIMO radar, this sparse MIMO system is better because its array aperture is 40, while the array aperture of ULA is 24.

It can be found from the simulation results that the sparse-array adopts fewer array elements to achieve the same DOA effect as the full array, while the MIMO system expands the array aperture, and thus improves the angular resolution. Therefore, the sparse-array and MIMO technology are both adopted in our system.

To further reduce the complexity of the system structure, a simplified signal transmission principle is proposed, and combined with the signal processing process, this will be introduced in the next section.

## 3. Sparse-Array TDM MIMO Radar System Signal Processing

### 3.1. Signal Model

**Figure 6:**
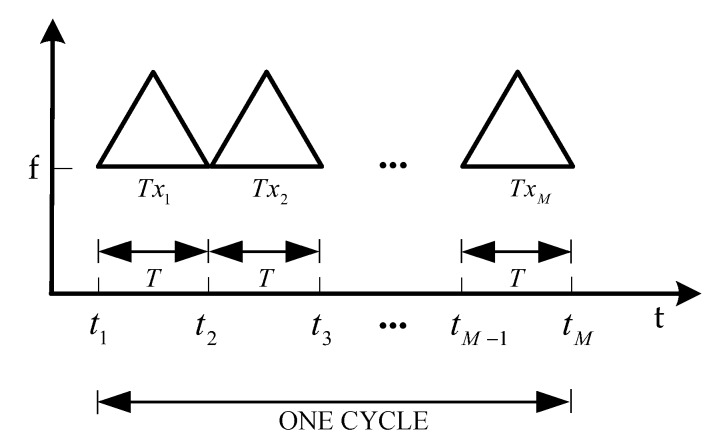
The transmission scheme of the system.

TDM transmission is an easy principle to simplify the structure of a radar system. The principle to achieve the TDM in the system is constantly activating the transmitters. Tx_1_ transmits a signal then Rx_1_ receives the echo signal before Tx_2_ activates, and in each time period only one transmitter works. The transmission scheme of this system is shown in Figure 6.

As seen in Figure 6, the system transmits the LFMCW signal, and the time duration of this LFMCW signal is T. During the working period of each transmitting antenna, the LFMCW signal of one cycle is transmitted. After M transmitting antennas work in turn, a whole cycle of data acquisition is obtained. The echo signal received by the receiving antenna through M timeslot is equivalent to the data received by the sparse linear array of M×G elements.

Taking the process of up sweep frequency as an example. The signal model of LFMCW [17] is given as:

(6) $$s_{t}(t)=U\exp\left[j2\pi \left(f_{0}t+\frac{kt^{2}}{2}\right)\right]$$

where U is the amplitude of the signal, k is the chirp rate of the up sweep frequency signal and k=2B/T.B is the bandwidth, and the echo signal can be expressed as follows:

(7) $$s_{r}(t)=U_{0}\exp\left\{j2\pi \left[f_{0}(t-\tau )+\frac{k{\left(t-\tau \right)}^{2}}{2}\right]\right\}$$

where $U_{0}$ is the amplitude of echo signal, τ is the delay time. Then zero intermediate frequency echo signal is obtained:

(8) $$s(t)=UU_{0}\exp\left\{j2\pi \left[f_{0}\tau +kt\tau -\frac{k\tau^{2}}{2}\right]\right\}$$

If the target speed is v, and its initial position is $R_{0}$, then the delay of the echo signal can be depicted as follows:

(9) $$\tau =\frac{2(R_{0}+vt)}{c}=\tau_{0}+\frac{2vt}{c}$$

where c is the velocity of light, and $\tau_{0}=2R_{0}/c$ is the echo delay of the stationary target. Substituting Formula (9) into (8), the zero intermediate frequency echo of LFMCW signal can be formulated as:

(10) $$s_{k}(t)=UU_{0}\exp\left\{j2\pi \left[\left(\frac{2f_{0}v}{c}+k\tau_{0}-\frac{2k\tau_{0}v}{c}\right)t+\left(\frac{2kv}{c}-\frac{2kv^{2}}{c^{2}}\right)t^{2}+f_{0}\tau_{0}-\frac{k\tau_{0}{}^{2}}{2}\right]\right\}$$

For the signal model of a sparse-array-based TDM MIMO radar system, let the first receiver Rx_1_ be as the reference element when Tx_1_ works. Its delay time between receiving and transmitting signals can be denoted as:

(11) $$\tau_{00}=\frac{2\left(R_{0}+vt\right)}{c}=\tau_{0}+\frac{2vt}{c}$$

Taking the case of the *m*th transmitter and *n*th receiver as an example. When Tx_m_ works, the time delay between the receiving and transmitting signals of Rx_n_ can be expressed as:

(12) $$\tau_{mn}=\frac{2[(R_{0}+vt)+mvT]}{c}+\frac{w_{mn}}{c}$$

where m=0,1,…,M−1, n=0,1,…,G−1, $w_{mn}=\left(m\cdot N\cdot d_{r}+n\cdot d_{rn}\right)\sin\theta$ and $w_{mn}/c$ is the time delay caused by the spacing the *m*th transmitter and *n*th receiver. Since v≪c, $w_{mn}\ll c$, the terms including ${\left(v/c\right)}^{2}$, ${\left(w_{mn}/c\right)}^{2}$ and $w_{mn}/c^{2}$ can all be ignored. Substituting Formula (12) into (8), the zero intermediate frequency echo of the system can be obtained as:

(13) $$s_{mn}(t)=UU_{0}\exp\left\{j2\pi \left[\begin{array}{l} \left(\frac{2f_{0}v}{c}+k\tau_{0}-\frac{2k\tau_{0}v}{c}+\frac{2kmvT}{c}\right)t+\\ \left(f_{0}-k\tau_{0}\right)\frac{2mvT}{c}+f_{0}\tau_{0}-\frac{k\tau_{0}{}^{2}}{2}+\frac{f_{0}w_{mn}}{c} \end{array}\right]\right\}$$

### 3.2. The Deviation Caused by Motion Targets

**Figure 7:**
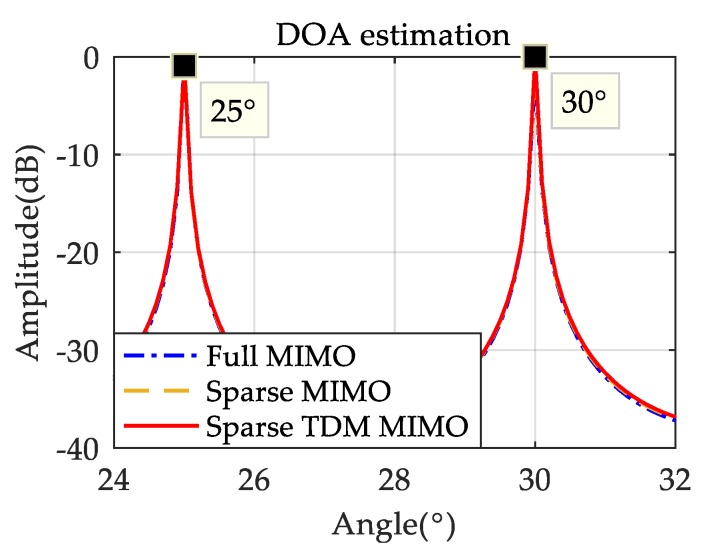
DOA performance for static targets.

This section is to verify the DOA performance of the sparse-array TDM MIMO system through simulation. The sparse-array optimized by the genetic algorithm in Section 2.2 is adopted as the receiver arrangement. In addition, the system uses a 24 GHz LFMCW signal, where SNR=10 dB, T=10 ms, B=100 MHz, sampling frequency $f_{s}=50\;\mathrm{KHz}$, and the two targets are static. The performance of the sparse-array MIMO system and of the full MIMO system are also simulated for comparison with the performance of the proposed system. Sparse-array MIMO radar adopts the same receiver arrangement as Figure 3b, and the full MIMO radar uses 2 Tx and 20 Rx antennas. The array apertures of the three systems are all 40. It can be seen from Figure 7 that the DOA performance of the sparse-array TDM MIMO radar is almost the same as that of the sparse-array MIMO radar and full MIMO radar when the targets are static.

**Figure 8:**
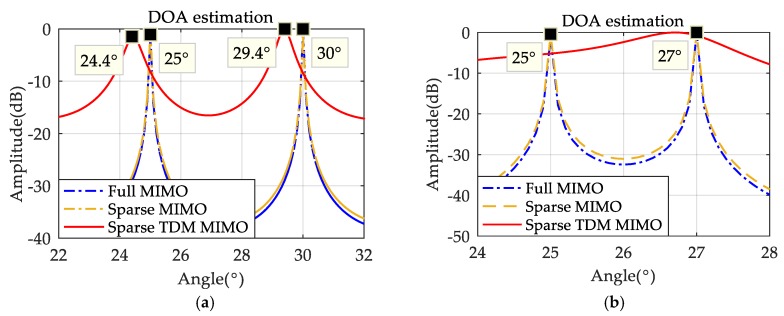
(**a**) DOA performance comparison for motion targets; (**b**) Angular resolution contrast of targets DOA estimation results for motion targets.

Next, the DOA performance of the system for detecting motion targets is simulated. The result is shown in Figure 8a, which shows that when detecting the target with high speed, the DOA estimation accuracy of the sparse TDM MIMO system is not as good as the ones of the sparse-array MIMO system and of the full MIMO system. Then the angle of the second target was changed to $\theta_{2}=27{}^{\circ}$ for another simulation, and the results are shown in Figure 8b. It can be found that when the targets get close, the DOA estimation performance of the sparse-array TDM MIMO system declines, and the adjacent targets cannot even be distinguished. This problem will be analyzed and solved in Section 3.3.

### 3.3. Signal Calibration Processing

**Figure 9:**
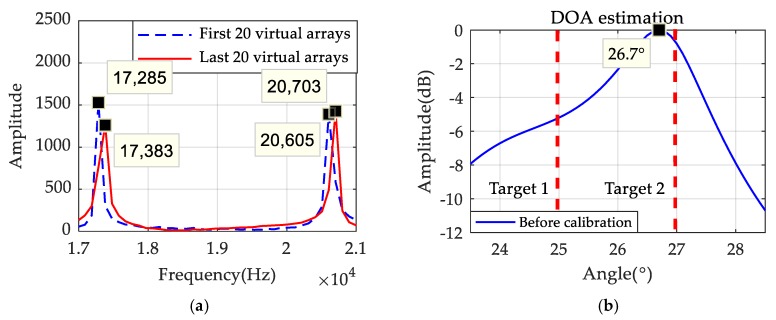
(**a**) FFT results of virtual arrays when targets in motion; (**b**) The DOA results before calibration.

Consider a static target, which means v=0; it can be found from Formulas (12) and (13) that the beat signal received by the virtual element is the same as the one of the traditional LFMCW MIMO radar system. Therefore, all virtual arrays can be equivalent to a sparse linear array that can be accurately used for DOA estimation. However, in case of the motion targets, which means that v≠0, the item of 2kmvTt/c in Formula (13) will cause a frequency migration. Assuming there are two targets, their angles are $\theta_{1}=25{}^{\circ}$ and $\theta_{2}=27{}^{\circ}$, respectively, and their moving speeds are $v_{1}=v_{2}=35m/s$. Other parameters are the same as mentioned above. By comparing the FFT results of the former 20 virtual arrays and the last 20 virtual arrays, which are shown in Figure 9a, frequency migration can obviously be found, and it will have impacts upon the results of the system DOA estimation. Figure 9b shows that the estimation results are affected by migration, and lead to the performance degradation of DOA estimation.

To correct the frequency migration, a method based on time stretching transform is presented. The migration term 2kmvTt/c can be eliminated. First, rewrite the Formula (13) as the following:

(14) $$\begin{array}{l} s_{mn}(t)=UU_{0}\exp\left\{j2\pi \left(f_{0}\tau_{0}-\frac{k\tau_{0}{}^{2}}{2}+\frac{f_{0}w_{mn}}{c}\right)\right\}\\ \times \exp\left\{j2\pi t\left(f_{d}+k\tau_{0}+kmT\frac{v}{c}\right)\right\}\\ \times \exp\left\{j2\pi f_{d}mT\right\} \end{array}$$

where $f_{d}=2vf_{0}/c$ is the Doppler Frequency, mT is the real slow time, and since $R_{0}\ll c$, the terms including $k\tau_{0}/c$ can be neglected when the target is moving at high speed; Doppler ambiguity will occur when the Doppler Frequency is more than half of the modulation frequency. Set the modulation frequency as F=1/T, and the ambiguous frequency as $f_{am}$; their relationship can be expressed as follows:

(15) $$f_{d}=f_{am}+rF$$

where r is a fuzzy factor. Taking the Doppler ambiguity into consideration and replacing $f_{d}$ with $f_{am}$, the Formula (14) can be rewritten as:

(16) $$\begin{array}{l} s_{mn}(t)=UU_{0}\exp\left\{j2\pi \left(f_{0}\tau_{0}-\frac{k\tau_{0}{}^{2}}{2}+\frac{w_{mn}}{c}\right)\right\}\\ \times \exp\left\{j2\pi t\left(f_{d}-rF+k\tau_{0}+kmT\frac{v}{c}\right)\right\}\\ \times \exp\left\{j2\pi mT(f_{d}-rF)\right\} \end{array}$$

defining a virtual slow time m'T, the transformation can be represented as:

(17) $$m=\frac{f_{0}}{f_{o}+kt}m'$$

substituting the transformation Formula (17) into (16) to get the following expression:

(18) $$\begin{array}{l} y_{mn}(t)=UU_{0}\exp\left\{j2\pi \left(f_{0}\tau_{0}-\frac{k\tau_{0}{}^{2}}{2}+\frac{f_{0}w_{mn}}{c}\right)\right\}\\ \times \exp\left\{-j2\pi \left(\frac{rFf_{0}}{f_{0}+kt}m'T+rFt\right)\right\}\\ \times \exp\left\{j2\pi t\left(k\tau_{0}+f_{d}\right)\right\}\\ \times \exp\left\{j2\pi f_{d}m'T\right\} \end{array}$$

from the above formula, it can be found that the transformed signal no longer contains the term that generates frequency migration. But the fuzzy term $-j2\pi \left(rFf_{0}m'T/\left(f_{0}+kt\right)+rFt\right)$ and phase error term $2\pi f_{d}m'T$ are still existing. The fuzzy term can be eliminated by multiplying the correction factor, which is shown in Formula (19).

(19) $$\begin{array}{l} y_{realmn}(t)=y_{mn}(t)\times \exp\left\{j2\pi \left(\frac{rFf_{0}}{f_{0}+kt}m'T+rFt\right)\right\}\\ =UU_{0}\exp\left\{j2\pi \left(f_{0}\tau_{0}-\frac{k\tau_{0}{}^{2}}{2}+\frac{f_{0}w_{mn}}{c}\right)\right\}\\ \times \exp\left\{j2\pi t\left(k\tau_{0}+f_{d}\right)\right\}\times \exp\left\{j2\pi f_{d}m'T\right\} \end{array}$$

Then, to handle the phase error, a simple and efficient method based on [22] is adopted in the system. After the echo signal is processed by FFT, the peaks of the spectrum will correspond to the frequency information of the targets. By searching the spectral peak, the corresponding position of the targets is determined, and several points near the targets are multiplied by the corresponding correction coefficient. The correction coefficient of the phase can be expressed as:

(20) $$\delta =e^{-j2\pi f_{d}mT}$$

Therefore, different phase errors can be eliminated separately, then the term $2\pi f_{d}m'T$ is removed. Finally, the frequency migration and phase error are eliminated. The process of system signal processing can be summarized as follows:

Step1. The frequency migration is corrected by a time stretching transformation of the signal.

Step2. The frequency migration corrected signal is multiplied by the fuzzy factor, and then the phase error correction is carried out to obtain the calibrated signal.

Step3. DOA estimation results are obtained by processing the calibrated signal with MUSIC.

**Figure 10:**
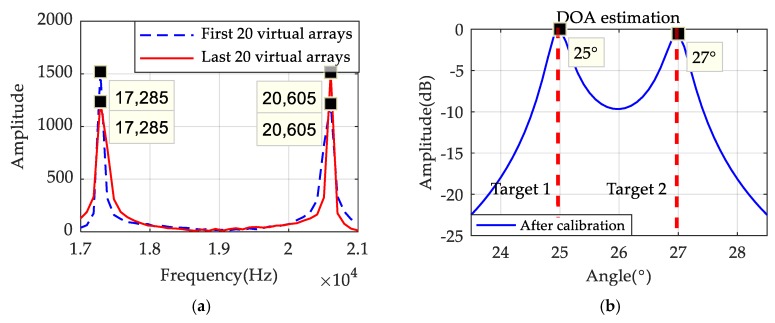
(**a**) The result of FFT after calibration; (**b**) The DOA results after calibration.

Simulations are carried out to observe whether the speed of the targets still affects the DOA result after the calibration. Assume there are two motion targets, and conditions are the same as mentioned above. The FFT results of half of the equivalent arrays are analyzed first. Figure 10a indicates the FFT results of the first, and the last equivalent array elements are consistent with each other, so there is no migration after the transformation method is taken. Figure 10b shows that two targets can be accurately detected when the signal is calibrated. The resolution and performance of DOA estimations are improved significantly with this method.

### 3.4. System DOA Performance after Calibration

**Figure 11:**
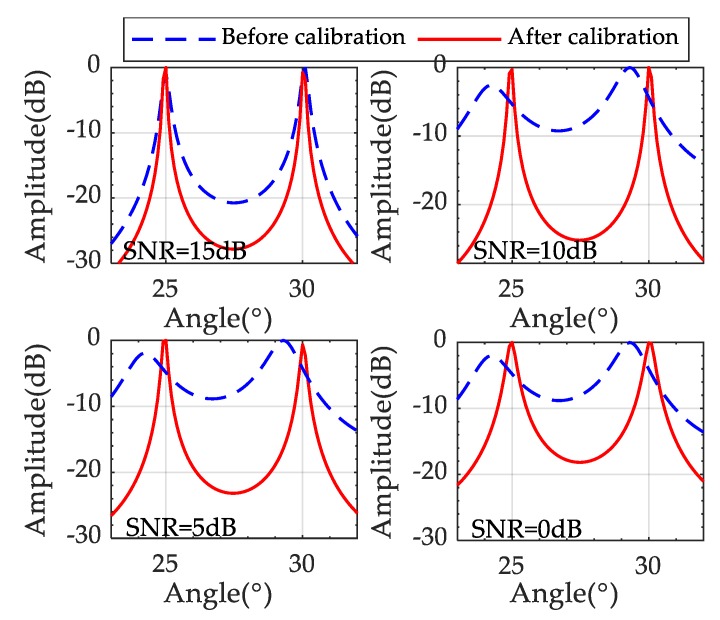
The DOA results at different SNR.

After calibration, the simulation of the sparse-array TDM MIMO radar system is carried out. Except for the velocity, angle, and SNR, the remaining conditions are the same as the previous simulation parameters. Setting the angles $\theta_{1}=25{}^{\circ}$, $\theta_{2}=30{}^{\circ}$, SNR1 = 15 dB, SNR2 = 10 dB, SNR3 = 5 dB, SNR4 = 0 dB and the targets velocities are $v_{1}=v_{2}=35m/s$. It can be seen from Figure 11 that the performance of DOA estimation is still good even under the low SNR condition after calibration.

**Figure 12:**
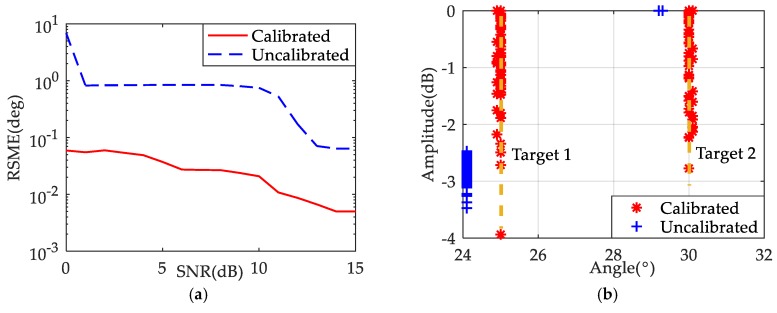
(**a**) The RSME of DOA results; (**b**) The DOA results of 100 Monte Carlo experiments.

Then the RSME of DOA estimation with SNR = 0–15 dB is simulated, the number of the Monte Carlo experiments is 100. It can be seen from Figure 12a that the RSME of the calibrated signal is lower than that of the uncalibrated signal, indicating that its DOA performance is better. Figure 12b is the result of a Monte Carlo experiment with SNR = 5 dB. It can be found that most DOA results of the calibration signal are close to the target position, while the uncalibrated signal deviates far from the correct target position and target 2 is missed many times.

## 4. Conclusions

In this paper, a sparse-array-based TDM MIMO radar system is proposed. By introducing a sparse-array, the structure of the system is simplified. Then the GA is used to optimize the sparse-array arrangement to achieve better performance of the system, which is indicated by the simulation results. The system transmits the TDM signal, which further simplifies the structure of the system. However, due to the characteristics of TDM, the system will generate frequency migration when detecting motion targets. To further improve system performance, the reason for the frequency migration is analyzed, and a method based on time stretching transformation is proposed to eliminate it. The calibrated signal no longer contains the frequency migration and phase error. Finally, comprehensive simulations were carried out to evaluate the performance of the system. Simulation results show the effectiveness of the calibration method, and the proposed system has good estimation performance, even in a low SNR environment.

However, the targets in this paper are assumed to be moving at a constant speed. If the speed of the target changes over time, further consideration should be taken into the calibration method. Besides, this paper assumes that the noise is white Gaussian, but typically, the radar system will be interfered with by disturbance events. Although the calibration method can still work, the DOA algorithm needs to be improved to deal with colored noise.
